# Epidemiological analysis of drinking water-type fluorosis areas and the impact of fluorosis on children’s health in the past 40 years in China

**DOI:** 10.1007/s10653-023-01772-9

**Published:** 2023-10-31

**Authors:** Feiqing Wang, Yanju Li, Dongxin Tang, Jianing Zhao, Bo Yang, Chike Zhang, Min Su, Zhixu He, Xiaodong Zhu, Dong Ming, Yang Liu

**Affiliations:** 1 Academy of Medical Engineering and Translational Medicine, Tianjin University, No. 92 Weijin Road, Nankai District, Tianjin City, 300072 China; 2 Clinical Research Center, The First Affiliated Hospital of Guizhou University of Traditional Chinese Medicine, No. 71 Bao Shan North Road, Yunyan District, Guiyang, 550001 Guizhou Province China; 3 Clinical Research Institute, Affiliated Hospital of Guizhou Medical University, Guiyang, 550004 Guizhou Province China; 4 National and Guizhou Joint Engineering Laboratory for Cell Engineering and Biomedicine Technique, Guiyang, 550004 Guizhou Province China; 5 Neurological Institute, Tianjin Medical University General Hospital, Tianjin, 300072 China

**Keywords:** Drinking water fluorosis, Defluoridation, Intelligence quotient, Health status, Fluorosis area

## Abstract

This study analyzed the effect of China’s fluorosis prevention and control program, which has been in effect for more than 40 years, and the impact of fluorosis on children’s health. Relevant research studies were retrieved from the following online databases from the time of their inception to May 2022: PubMed, ScienceDirect, Embase, Cochrane, China National Knowledge Infrastructure, and Wanfang. The Review Manager 5.3 software was used in statistical analyses. This article included seventy studies: Thirty-eight studies reported the effect of improving water quality and reducing fluoride content, the incidence rate of dental fluorosis in children, and the level of urinary fluoride, and thirty-two studies reported the intelligence quotient (IQ) and health status of children. Following water improvement strategies, the fluoride levels in drinking water decreased significantly; urinary fluoride levels and dental fluorosis decreased significantly in children. With regard to the effect of fluorosis on the IQ of children, the results showed that the IQ of children in areas with a high fluoride of fluorosis was lesser than that in areas with a low fluoride, and this difference was significant. Based on the prevalence of dental fluorosis and its effect on the intelligence of children, it appears that reducing fluoride levels in drinking water and monitoring water quality are important strategies for the prevention and treatment of fluorosis.

**Graphical abstract:**

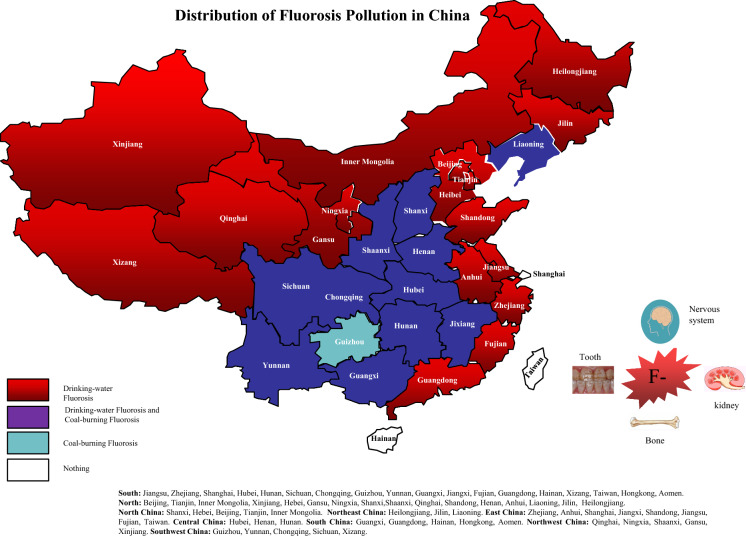

**Supplementary Information:**

The online version contains supplementary material available at 10.1007/s10653-023-01772-9.

## Introduction

According to the World Health Organization (WHO), fluorosis has become a major health problem worldwide, as over 260 million people globally are drinking water from sources with high-fluoride concentrations (Amini et al., 2008). Drinking water is the most important pathway of fluoride intake, as it accounts for 75–90% of fluoride intake (Habtamu et al., 2019; Kloos & Haimanot, 1999). China has the highest prevalence of fluorosis and faces the most serious harmful effects of fluorosis in the world. This is because China is located in the fluoride belt. Excess fluoride levels have been detected in the groundwater in areas of China where the fluoride content is high in rocks or soil. Fluorosis caused by the consumption of drinking water with high-fluoride content, which will henceforth be referred to as drinking water fluorosis, is one of the most important types of endemic fluorosis in China, and it is prevalent in 27 provinces, cities, and autonomous regions across the North and South of China (Li et al., 2020a, 2020b, 2020c; Wang et al., 2012a, 2012b).

The severity of fluorosis is associated with the concentration of fluoride in drinking water, daily intake, duration of exposure, and climatic conditions (Li et al., 2021). Exposure to high-fluoride levels for a prolonged period results in dental fluorosis, skeletal fluorosis, and non-skeletal fluorosis, which manifests as muscle weakness, tiredness, anemia, dyspepsia, male infertility, decrease in IQ, and other symptoms (Everett, 2011; Kumar et al., 2009; Shashi et al., 2008; Wei et al., 2019). Epidemiological investigations have found that fluorosis can severely affect any age group and can range from mild dental fluorosis to crippling skeletal fluorosis. Its effects are especially harmful to developing children, and its adverse effects are irreversible (Alexandra et al., 2021; Alvee et al., 2021). In 1937, Murray first reported the manifestation of nervous system dysfunction in patients with endemic fluorosis (Murray, 1937). Since then, other studies have found that long-term excessive intake of fluoride can cause demyelinating changes in the cerebral cortex and subcortical areas and lead to hypothyroidism; this may explain the decline in the intelligence level of children in high-fluoride areas (Fan, 2020; Saeed et al., 2020; Wang et al., 2021a, 2021b, 2021c). As a result of these findings, and the fact that approximately 100 million people in 1115 counties are presently affected by fluoride threats in China (Wang et al., 2020), the functional and organic effects of fluorosis on the central nervous system, especially on children’s intellectual development, learning, and memory, have been receiving increasing attention.

The most effective way to prevent and treat fluorosis is to improve water quality and reduce water fluoride content (James et al., 2021; Wang et al., 2021a, 2021b, 2021c). So far, there is no effective clinical treatment for endemic fluorosis, and reducing fluoride exposure level and reducing fluoride intake are the main measures adopted by most countries (Malin et al., 2019; Zhao et al. 2013; Mohd Nor et al., 2021). In China, since 1978, fluoride-safe drinking water supply schemes have been implemented to provide fluoride-safe drinking water to populations in high-fluoride areas and fluorosis endemic areas (Li et al., 2020a, 2020b, 2020c). Through decades of effort on the part of the government, the fluoride concentrations in the drinking water supplied to many villages have been under control. However, due to differences in the implementation periods and management strategies, there are still significant differences in the effects of these water improvement and defluoridation projects among various provinces. At present, the effects of the water improvement and fluoride reduction projects that have been implemented in China for more than half a century have not been systematically analyzed and studied. Therefore, this study was undertaken to comprehensively assess the effects of these projects on the prevention and treatment of drinking water-borne endemic fluorosis and the effects of fluorosis on the development and intelligence levels of children. These findings are important from the perspective of monitoring the effects of these projects and highlighting potential areas of improvement.

## Methods

### Literature retrieval strategy

The PubMed, ScienceDirect, Embase, Cochrane, China National Knowledge Infrastructure (CNKI), and Wanfang databases were searched for relevant articles deposited from their inception to May 2022, using the following search terms in the title, keyword, or abstract: water improvement, defluorination, dental fluorosis, skeletal fluorosis, children, student, intelligence, intelligence quotient, nervous system, and behavior. Additionally, we performed a backward literature search by manually retrieving the references included in the studies and related reviews.

### Inclusion and exclusion criteria

Three investigators independently performed the data extraction. This study evaluated the content and scope of previously published reports and generated inclusion and exclusion criteria. The following were the inclusion criteria: publication from the inception of each database to May 2022, which belongs to the exposure group (fluoride content in drinking water > 1.0 mg/L), dental fluorosis in children, skeletal fluorosis in adults, and data on urinary fluoride levels in children and/or adults. Combined Raven’s test (CRT) measures a child’s IQ (Julvez et al., 2021; Theobald et al., 2018). Based on the IQ level, the data were divided into seven categories: very excellent (IQ > 130), excellent (IQ = 120–129), upper middle (IQ = 110–119), medium (IQ = 90–109), lower middle (IQ = 80–89), marginal (IQ = 70–79), and mental retardation (IQ < 69). The main advantage of the combined Raven test is that it assesses the basic factors that represent children’s intelligence structure, and it is therefore suitable for collective testing.

### Data analysis

This research evaluated the study content and scope of previously published reports. A list of relevant data from each study was created, including publication time, type of study, time and place of investigation, duration of water treatment, prevalence of dental fluorosis in children between the ages of 8 and 15 years, prevalence of skeletal fluorosis in adults, fluoride levels in drinking water, fluoride levels in urine and child’s IQ, etc. When there was heterogeneity, sensitivity analysis or appropriate statistical methods were used to estimate the effect. The heterogeneity of the research indicators was analyzed to determine whether the indicators could be pooled for analysis. The data included.

### Statistical analysis

All statistical analyses were completed utilizing the Review Manager version 5.3. The chi-square test (χ^2^) was used to evaluate heterogeneity among the studies (*P* = 0.05). Heterogeneity among studies was assessed using the I^2^ statistic. 25%, 50%, and 75% were designated as low, medium, and high for I^2^ values, respectively. We performed the study using a fixed-effect model, where with I^2^ > 50% we used random-effect model. Weighted mean difference (WMD) and standardized mean difference (SMD) were used for continuous variables, and odds ratio (OR) was used for binary variables and reported pooled estimates and the corresponding 95% confidence interval (CI). Study quality assessment and risk of bias were assessed for all studies. In the study, differences were considered statistically significant when the *p* value was ≤ 0.05.

## Results

### Literature search results

**Fig. 1:**
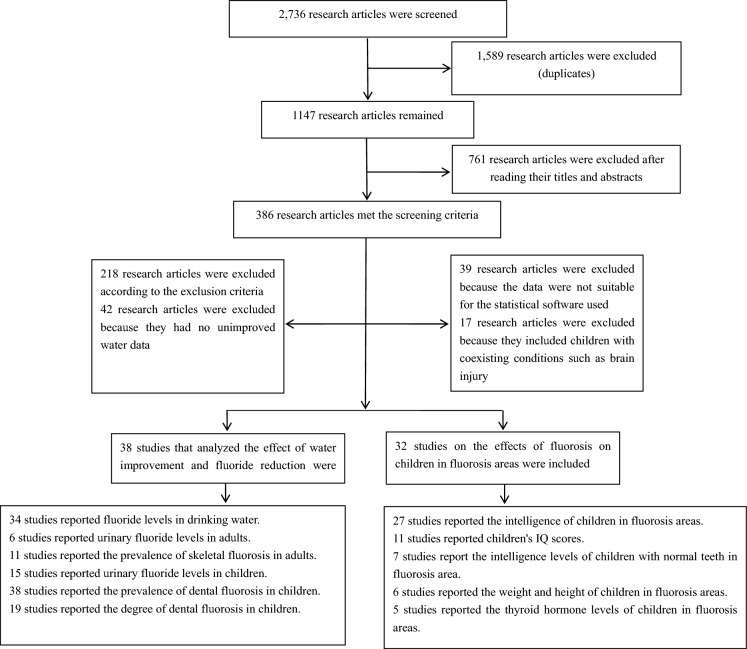
Flowchart depicting the study selection process

Out of a total of 2736 studies that were retrieved, 119 were obtained from PubMed, ScienceDirect, Embase, and Cochrane and 2617 were obtained from CNKI and Wanfang. Out of the 2736 studies, 1589 were duplicates: 57 were included in PubMed, ScienceDirect, Embase, and Cochrane, and 329 were included in CNKI and Wanfang. After application of the exclusion criteria, a total of seventy studies were finally included in the meta-analysis: thirty-eight studies reported improvement in the incidence of fluorosis and thirty-two studies reported on children’s intelligence levels in fluorosis areas (Fig. 1).

The seventy included research articles were cross-sectional studies published between 1995 and 2021. Thirty-eight studies reported on water improvement projects that were carried out over a period of 5–40 years and covered 22 provinces, cities, and counties. Thirty-two studies reported on children’s intelligence levels in fluorosis areas and covered 14 provinces, cities, and counties. Thirty-eight of the studies reported the prevalence of dental fluorosis in children in 32,042 cases, and thirty-four studies reported the fluoride levels in drinking water (Table S1). Twenty-seven studies reported intelligence levels in 14,617 children in fluorosis areas, and eleven studies report children’s IQ scores (Table S1).

### Epidemiological analysis of reducing water fluoride content in fluorosis areas

Thirty-four studies (Table S1) reported fluoride levels in drinking water, and the results showed that the fluoride content in water decreased significantly (95% CI − 2.72 to − 1.96, *P* < 0.05; Table 1). For subgroup analysis, the studies were divided into those from South China (95% CI − 4.69 to − 1.55) and North China (95% CI − 2.38 to − 1.81), and the studies were further divided into six areas of China: North China (95% CI − 2.86, − 1.49), Northeast China (95% CI − 2.10 to − 0.68), East China (95% CI 3.26 to − 1.81), Central China (95% CI − 8.01 to 0.25), South China (95% CI − 2.97 to − 1.49), and Northwest China (95% CI − 3.59 to − 1.02), and the fluoride content in water decreased significantly (all *P* < 0.05; Table 1). Subgroup analysis was also conducted according to the duration of the water improvement: 0–10 years (95% CI − 1.95 to 1.29), 11–20 years (95% CI − 7.17 to − 1.37), and 21–30 years (95% CI − 2.82 to − 1.61), the differences were statistically significant (*P* < 0.05; Table 1).

**Table 1:** Comparison of water improvement and fluoride reduction among in different areas

Area	Test for heterogeneity		Analysis model	Test for overall effect		WMD or SMD	95% CI
	*I*^2^ (%)	*P*		*Z*	*P*		
Total	100	< 0.00001	Random	12.02	< 0.00001	− 2.34	(− 2.72, − 1.96)
Two areas							
South	100	< 0.00001	Random	3.89	= 0.0001	− 3.12	(− 4.69, − 1.55)
North	99	< 0.00001	Random	12.31	< 0.00001	− 2.09	(− 2.38, − 1.81)
Six areas							
North China	99	< 0.00001	Random	6.23	< 0.00001	− 2.17	(− 2.86, − 1.49)
Northeast China	98	< 0.00001	Random	3.85	= 0.0001	− 1.39	(− 2.10, − 0.68)
East China	99	< 0.00001	Random	6.85	< 0.00001	− 2.53	(− 3.26, − 1.81)
Central China	100	< 0.00001	Random	1.84	*P* = 0.07	− 3.88	(− 8.01, 0.25)
South China	93	< 0.00001	Random	5.89	< 0.00001	− 2.23	(− 2.97, − 1.49)
Northwest China	99	< 0.00001	Random	3.51	= 0.0004	− 2.31	(− 3.59, − 1.02)
Time							
0–10 Years	100	< 0.00001	Random	9.54	< 0.00001	− 1.62	(− 1.95, 1.29)
11–20 Years	100	< 0.00001	Random	2.89	= 0.004	− 4.27	(− 7.17, − 1.37)
21–30 Years	98	< 0.00001	Random	7.13	< 0.00001	− 2.22	(− 2.82, − 1.61)

*WMD* weighted mean difference, *SMD* standardized mean difference, *95% CI* 95% confidence intervals

### Epidemiological analysis of urinary fluoride levels and incidence of skeletal fluorosis in adults after water improvement

**Fig. 2:**
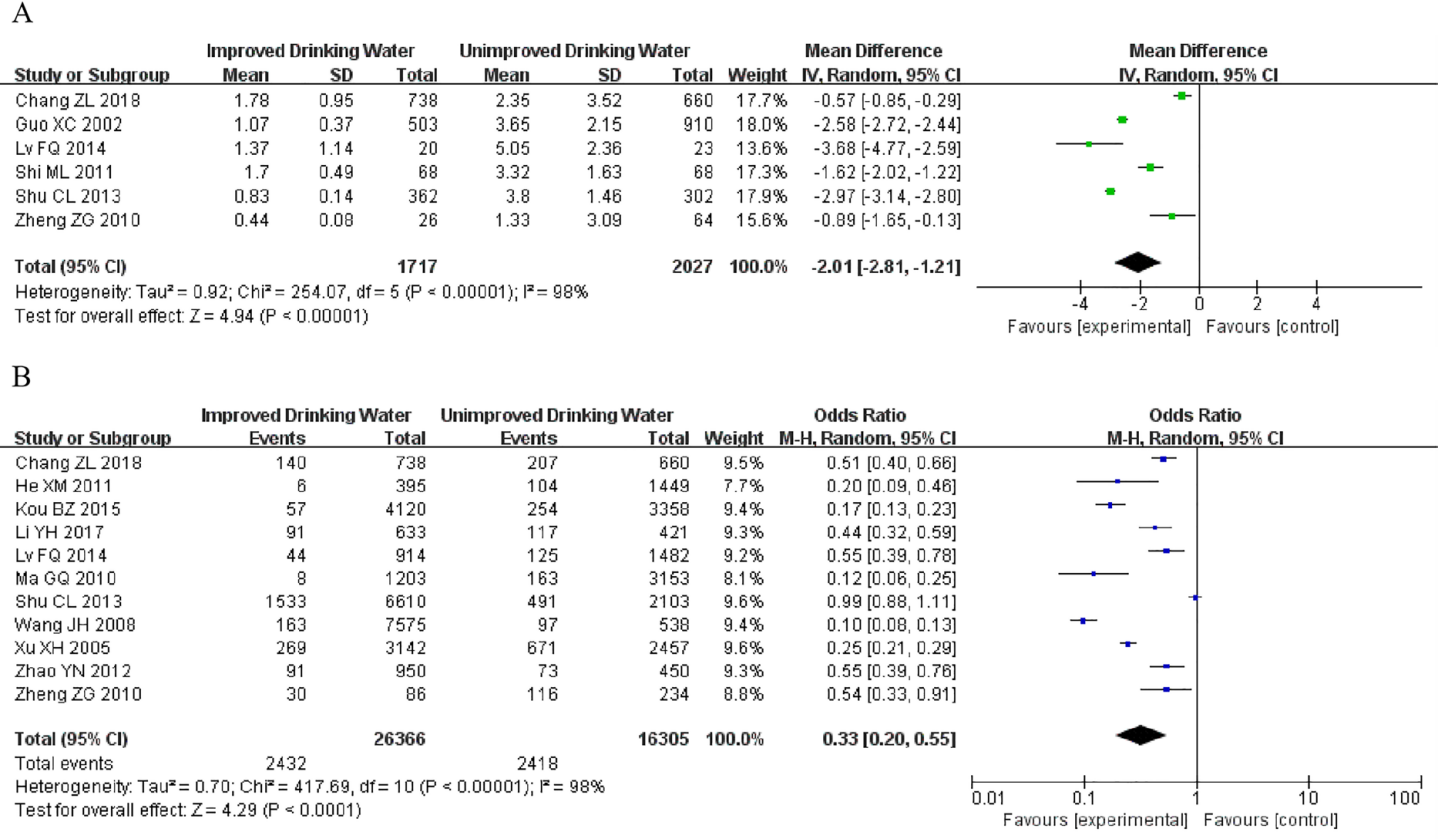
Urinary fluoride levels and incidence of skeletal fluorosis in adults. **A** Urinary fluoride levels in adults. **B** Prevalence of skeletal fluorosis in adults

Six studies (Table S1) reported urinary fluoride levels in adults. The research findings showed (WMD =  − 2.01, 95% CI − 2.81 to − 1.21, *P* < 0.00001) there was a statistically significant decrease in urinary fluoride levels (*P* < 0.05; Fig. 2). Eleven studies (Table S1) reported the prevalence of skeletal fluorosis in adults. The research findings showed (OR = 0.33, 95% CI 0.20–0.55, *P* < 0.0001) a statistically significant decrease in skeletal fluorosis (*P* < 0.05; Fig. 2).

### Epidemiological analysis of dental fluorosis prevalence in children after water improvement

Thirty-eight studies (Table S1) reported data on dental fluorosis in children. These studies also showed (OR = 0.13, 95% CI 0.10–0.18, *P* < 0.00001) a significant difference in the prevalence of decreased dental fluorosis in children (*P* < 0.05; Table 2). Using subgroup analysis, the following results were obtained: South China (95% CI 0.06–0.14) and North China (95% CI 0.11–0.23), and the differences between the two regions were statistically significant (*P* < 0.05; Table 2). The studies were further divided into six areas: North China (95% CI 0.15–0.41), Northeast China (95% CI 0.10–0.35), East China (95% CI 0.05–0.19), Central China (95% CI 0.04–0.18), South China (95% CI 0.01–0.38), and Northwest China (95% CI 0.03–0.44) (all *P* < 0.05; Table 2). The studies were divided based on the duration of the water improvement projects into 0–10, 11–20, and 21–30 years, and the differences between the three groups were statistically significant (all *P* < 0.05; Table 2). Nineteen studies (Table S1) reported the degree of dental fluorosis in children after water improvement. The studies showed (OR = 0.65, 95% CI 0.51–0.82, *P* = 0.0004) a statistically significant damage decrease in the degree of dental fluorosis in children (*P* < 0.05; Table 2).

**Table 2:** Comparison of the prevalence of dental fluorosis in children among in different areas

Area	Test for heterogeneity		Analysis model	Test for overall effect		Odds ratio	95% CI	Unimproved	Improved
	*I*^2^ (%)	*P*		*Z*	*P*			Total/events	Total/events
Total	99	< 0.00001	Random	12.63	< 0.00001	0.13	(0.10, 0.18)	96,442/52217	121,699/25918
Two areas									
North	97	< 0.00001	Random	11.05	< 0.00001	0.09	(0.06, 0.14)	48,553/22452	111,212/23385
South	99	< 0.00001	Random	9.25	< 0.00001	0.16	(0.11, 0.23)	47,889/29765	10,487/2533
Six areas									
North China	99	< 0.00001	Random	5.26	< 0.00001	0.24	(0.15, 0.41)	26,122/12658	54,084/17912
Northeast China	98	< 0.00001	Random	5.34	< 0.00001	0.19	(0.10, 0.35)	11,276/2381	42,619/2892
East China	96	< 0.00001	Random	7.11	< 0.00001	0.10	(0.05, 0.19)	18,765/10176	7912/1664
Central China	94	< 0.00001	Random	6.23	< 0.00001	0.08	(0.04, 0.18)	2677/1985	1720/404
South China	85	= 0.001	Random	3.05	= 0.002	0.07	(0.01, 0.38)	27,572/18337	1968/641
Northwest China	100	< 0.00001	Random	3.11	= 0.002	0.11	(0.03, 0.44)	10,030/6680	13,396/2405
Time									
0–10 Years	98	< 0.00001	Random	8.10	< 0.00001	0.22	(0.15, 0.32)	23,964/10715	33,636/5423
11–20 Years	100	< 0.00001	Random	4.04	< 0.0001	0.11	(0.04, 0.32)	41,689/27056	38,028/15426
21–30 Years	99	< 0.00001	Random	4.47	< 0.00001	0.07	(0.02, 0.22)	24,437/11934	11,847/1951
Degree of dental fluorosis in children									
Total	98	< 0.00001	Random	3.54	= 0.0004	0.65	(0.51, 0.82)	154,932/25823	316,962/52974
Normal	96	< 0.00001	Random	10.16	< 0.00001	5.07	(3.71, 6.94)	25,822/12156	52,827/43583
Suspect	97	< 0.00001	Random	1.19	= 0.23	1.30	(0.84, 2.02)	25,822/2437	52,827/4389
Very mild	93	< 0.00001	Random	3.37	= 0.0007	0.61	(0.46, 0.81)	25,822/5290	52,827/3408
Mild	86	< 0.00001	Random	8.25	< 0.00001	0.29	(0.22, 0.39)	25,822/3477	52,827/1240
Moderate	77	< 0.00001	Random	8.26	< 0.00001	0.16	(0.10, 0.25)	25,822/1725	52,827/298
Severe	87	< 0.00001	Random	5.39	< 0.00001	0.05	(0.02, 0.14)	25,822/738	52,827/56

*95% CI* 95% confidence intervals

### Epidemiological analysis of Urinary fluoride levels in children after water improvement

**Fig. 3:**
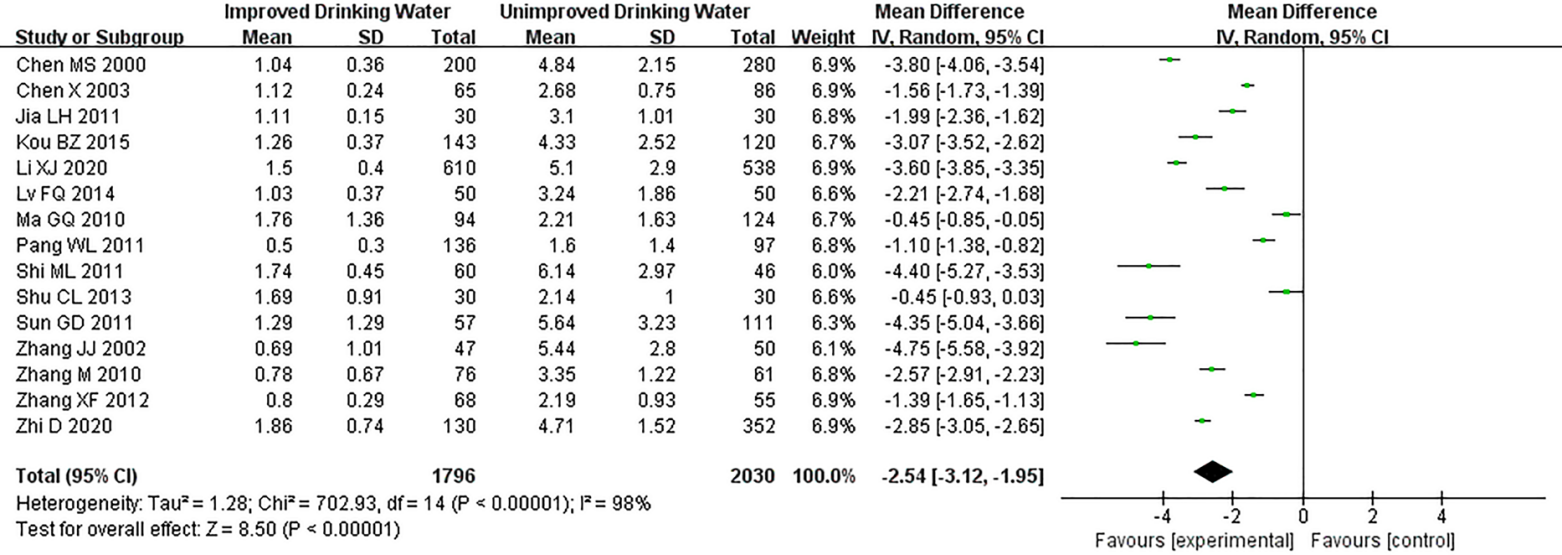
Urinary fluoride levels in children

Fifteen studies (Table S1) reported urinary fluoride levels, and the research findings showed (WMD =  − 2.54, 95% CI − 3.12 to − 1.95, *P* < 0.00001) a statistically significant decrease in urinary fluoride levels in children after implementation of the water improvement (*P* < 0.05; Fig. 3).

### Epidemiological analysis of IQ level in children in fluorosis areas

Twenty-seven studies (Table S1) reported IQ levels in children in normal areas (n = 7133) and fluorosis areas (n = 7484). The results showed children living in fluorosis areas had significantly lower IQ levels than children living in normal areas (*P* < 0.05, 95% CI − 8.95 to − 5.16, Table 3). For subgroup analysis, the studies were divided into mild-to-moderate and severe fluorosis areas, for which the following results were obtained: mild-to-moderate fluorosis areas: WMD = 6.64, 95% CI − 9.04 to − 4.24, *P* < 0.00001, and severe fluorosis areas: WMD =  − 7.47, 95% CI − 10.20 to − 4.75, *P* < 0.00001. The differences between the fluorosis and normal areas were statistically significant (*P* < 0.05; Table 3). The studies were also divided into those reporting drinking water-borne fluorosis and coal burning-associated fluorosis, for which the results were WMD =  − 6.11, *P* < 0.00001 and WMD =  − 13.59, *P* < 0.00001, respectively. The differences between the groups were statistically significant (*P* < 0.05; Table 3).

**Table 3:** Comparison of IQ level children in fluorosis area and normal area

Class	Test for heterogeneity		Analysis model	Test for overall effect		WMD or SMD	95% CI
	*I*^2^ (%)	*P*		*Z*	*P*		
Total	94	< 0.00001	Random	7.29	< 0.00001	− 7.06	(− 8.95, − 5.16)
Fluorosis area							
Mild to moderate	95	< 0.00001	Random	5.43	< 0.00001	− 6.64	(− 9.04, − 4.24)
Severe	91	< 0.00001	Random	5.38	< 0.00001	− 7.47	(− 10.20, − 4.75)
Type							
Drinking water	93	< 0.00001	Random	6.93	< 0.00001	− 6.11	(− 7.84, − 4.38)
Coal burning	74	= 0.009	Random	5.56	< 0.00001	− 13.59	(− 18.38, − 8.80)

*WMD* weighted mean difference, *SMD* standardized mean difference, *95% CI* 95% confidence intervals

### Analysis of the IQ scores of children in fluorosis areas

Eleven studies (Table S1) reported the degree of IQ in children. When the number of children under each IQ category was compared between children in normal areas (n = 3361) and children in severe fluorosis areas (n = 4027), a significantly lower number of children from normal areas fell under the lower IQ categories. However, a significantly higher number of children from normal areas than fluorosis areas fell under the higher IQ categories (*P* < 0.00001; Table 4).

**Table 4:** Comparison of IQ scores of children in fluorosis area and normal area

IQ class	Test for heterogeneity		Analysis model	Test for overall effect		Odds ratio	95% CI	Fluorosis	Normal
	*I*^2^ (%)	*P*		*Z*	*P*			Total/events	Total/events
< 69	0	= 0.65	Fixed	6.56	< 0.00001	3.02	(2.17, 4.20)	4027/213	3361/45
70–79	41	= 0.07	Fixed	1.35	= 0.0007	1.35	(1.09, 1.69)	4027/276	3361/135
80–89	27	= 0.19	Fixed	2.48	= 0.01	1.22	(1.04, 1.43)	4027/498	3361/303
90–109	49	= 0.03	Fixed	0.94	= 0.35	0.96	(0.87, 1.05)	4027/1759	3361/1520
110–119	24	= 0.21	Fixed	0.18	= 0.86	0.99	(0.88, 1.11)	4027/806	3361/744
120–129	30	= 0.16	Fixed	2.69	= 0.007	0.81	(0.70, 0.94)	4027/389	3361/434
> 130	0	= 0.75	Fixed	6.13	< 0.00001	0.44	(0.34, 0.57)	4027/86	3361/180

*95% CI* 95% confidence intervals

### Analysis of IQ in children with dental fluorosis in fluorosis areas

**Fig. 4:**
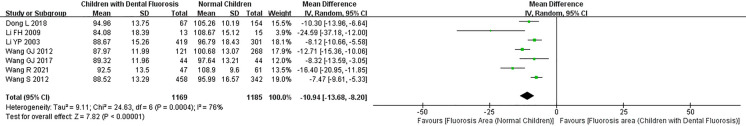
IQ levels in children with normal teeth and dental fluorosis in fluorosis areas

Seven studies (Table S1) reported the IQ levels in children with normal dentition (n = 1185) and dental fluorosis (n = 1169) in fluorosis areas. The results showed there was a statistically significant decrease in IQ levels between children with dental fluorosis and those with normal dentition in fluorosis areas (*P* < 0.05; 95% CI − 13.68 to − 8.20, Fig. 4). Additionally, three studies (Table S1) reported the degree of IQ in the two groups of children, and statistically significant differences were found for all IQ levels (*P* < 0.05; Table S2).

### Effects of fluorosis on thyroid hormone levels, language IQ, weight, and height

Five studies (Table S1) reported thyroid hormones levels in children from normal areas and fluorosis areas. Significant differences were found for TSH (*P* = 0.009, 95% CI 0.29–1.99), T4 (*P* < 0.0001, 95% CI − 4.30 to − 3.09), and FT4 (*P* < 0.0001, 95% CI − 1.54 to − 0.54), but no significant differences were found for T3 (*P* = 0.52) and FT3 (*P* = 0.81) (Table 5). Two studies (Table S1) reported language IQ and performance IQ in children from normal areas and fluorosis areas, and significant differences were found for all categories (Table 5). Six studies (Table S1) reported height and weight in children from normal areas and fluorosis areas, but there was no statistically significant difference between the two groups.

**Table 5:** Analysis of thyroid hormone level and language IQ of children in fluorosis area

IQ class	Test for heterogeneity		Analysis model	Test for overall effect		WMD or SMD	95% CI
	*I*^2^ (%)	*P*		*Z*	*P*		
Thyroid hormones							
TSH	97	< 0.00001	Random	2.62	= 0.009	1.14	(0.29, 1.99)
T3	81	= 0.005	Random	0.64	= 0.52	0.08	(− 0.16, 0.32)
T4	0	= 0.82	Random	11.97	< 0.00001	− 3.70	(− 4.30, − 3.09)
FT3	73	= 0.05	Random	0.24	= 0.81	0.05	(− 0.36, 0.46)
FT4	0	= 0.60	Random	4.09	< 0.0001	− 1.04	(− 1.54, − 0.54)
Language IQ performance IQ							
Common sense	0	= 0.59	Random	5.33	< 0.00001	− 2.62	(− 3.59, − 1.66)
Similar	80	= 0.03	Random	3.27	= 0.001	− 4.41	(− 7.05, − 1.77)
Arithmetic	65	= 0.09	Random	5.28	< 0.00001	− 5.70	(− 7.81, − 3.58)
Vocabulary	0	= 0.51	Random	8.66	< 0.00001	− 4.21	(− 5.17, – − 3.26)
Comprehension	80	= 0.03	Random	9.41	< 0.0001	− 3.34	(− 4.94, − 1.73)

*WMD* weighted mean difference, *SMD* standardized mean difference, *95% CI* 95% confidence intervals

## Discussion

This is the first study to retrospectively analyze the relationship between water fluoride content, urine fluoride, dental fluorosis, project duration, fluorosis incidence, and children’s health and IQ in fluorosis areas after the implementation of China’s water improvement and defluoridation project. The findings provide important insight into the impact of these programs on the incidence of fluorosis and the effect of fluorosis on the intelligence level of children. They also highlight some areas of the project that need attention in the future.

The present data show that there was a significant reduction in water fluoride levels following the implementation of the water improvement and defluoridation projects in different provinces in China, which is in accordance with WHO standards. The findings showed that the fluoride content of water in the South was higher than that in the North. After water improvement and defluoridation, the fluoride content of water in the South was lower than that in the North. According to the results for different districts, the water fluoride content in Central China was the highest, and it was followed in descending order by East China, North China, Northwest China, and South China. The water fluoride content in Northeast China was the lowest. This study found that while there were significant regional differences in the fluoride content of drinking water in China (Wu et al., 2021), the water improvement and defluoridation project effectively reduced the fluoride content in drinking water across all regions.

Skeletal fluorosis mostly occurs in young adults (Meena & Gupta, 2021; Srivastava & Flora, 2020). It is a chronic invasive systemic bone disease caused by long-term excessive intake of fluoride and is an important indicator of the effect of water improvement and defluoridation strategies (WHO, 2014; Idowu et al., 2019). The present results showed that the prevalence of skeletal fluorosis and urinary fluoride levels in adults and children decreased significantly after implementation of the water improvement projects in China. The findings of this study indicate that the fluoride content in the human body can be effectively reduced by water improvement and defluoridation strategies.

Dental fluorosis in children showed that water improvement strategies decreased significantly. The results showed that the incidence rate of dental fluorosis in children was higher in the South than that in the North, and this is consistent with the trend in water fluoride content. This implies that the incidence rate of dental fluorosis in children was positively correlated with the water fluoride content. The decrease in the incidence of dental fluorosis among children was most pronounced in Northeast China. According to Dean’s fluorosis index, the findings of the current analysis indicate that the severity of dental fluorosis in children was significantly reduced following the implementation of the water improvement and defluoridation project, with the percentage of children classified as normal increasing significantly. Overall, the water improvement and fluoride reduction project in China has been implemented for more than 40 years and has resulted in a 30% decrease in the incidence of dental fluorosis in children.

Long-term excessive intake of fluoride does not only cause extensive damage to hard tissues such as teeth and bones, but also causes extensive damage to soft tissues to varying degrees (Niu et al., 2018; Quadri et al., 2018; Riddell et al., 2019; Sabine et al., 2020; Zhou et al., 2015). Most studies have reported that the IQ of children with long-term high-fluoride intake was significantly lower than that of children with normal fluoride intake (Dec et al., 2017; Lee et al., 2016; Gong and James 2020). On the contrary, a study conducted in New Zealand concluded that there was no evidence to indicate that fluoride exposure affects the neurologic development or IQ of children (Broadbent et al., 2015). Therefore, there is some controversy about the influence of fluorosis on children’s intelligence. Our results showed that the IQ of children in fluorosis areas was lower than that of children in normal areas. There was a negative correlation between children’s IQ and fluoride intake, with the IQ of children in high-fluoride areas lower than that of children in low fluoride areas, IQ of children with dental fluorosis lower than that of children with normal teeth in fluorosis areas, and IQ of children related to the source of fluorosis. The study found that there were significant differences in the IQ scores between normal areas and fluorosis areas, with a significantly higher percentage of children in the fluorosis areas having IQ scores below 89 and a significantly higher percentage of children in normal areas having IQ scores higher than 120. In addition, the IQ scores of children with dental fluorosis were significantly lower than those of children with normal teeth. This has important implications, as this has been a controversial issue in the literature. Thus, these findings further emphasize the impact of fluoride exposure on intelligence levels in children.

So far, thyroid hormone levels have been identified as markers of mental retardation in children exposed to fluoride (Broadbent et al., 2015; Xu et al., 2020). Our results showed that the TSH level of children in fluorosis areas was significantly higher than that in normal areas, and the T4, FT4, and language IQ levels were significantly lower than those in normal areas. This is consistent with the results of Hara (1980).

The provinces this study have included are relatively large and time long, which results in significant heterogeneity in the analysis results. Through sensitivity and subgroup analysis, it was found that heterogeneity had a small impact on the results of this study. This study is the first to systematically analyze the effect of water quality improvement and fluoride reduction strategies in China over the last 40 years. The findings confirm all the reports so far that water improvement and fluoride reduction are effective measures to prevent endemic fluorosis caused by drinking water and can also effectively reduce the impact of fluorosis on children’s intelligence. However, the findings also point to some weak links and problems that need to be resolved in this regard. Due to the limited service life of water improvement facilities, poor management, and inadequate health supervision, a considerable number of water improvement and fluoride reduction projects have been discontinued or scrapped altogether. However, it is necessary to continue to invest in these projects in fluorosis endemic areas and strengthen the management and maintenance of the fluoride reduction and water improvement project, in order to ensure that residents of these areas have access to drinking water with low fluoride content. This will help to effectively control the disease and continue to reap the benefits of the prevention and control measures that have been in place over the last few decades.

## Conclusions

This is the first retrospective analysis of the effect of the water improvement and fluoride reduction project in China, which has been implemented over the past 40 years. The findings demonstrate that the water fluoride content, the incidence of dental fluorosis, and the incidence of skeletal fluorosis were effectively reduced as a result of this project. The findings also highlighted obvious regional differences in fluorosis areas, as the water fluoride content and the incidence rate of dental fluorosis were significantly higher in the South than in the North. This implies that efforts for the prevention and control of fluorosis in the South should be further strengthened. The present results also suggest that fluorosis can cause a reduction in children’s IQ, and this effect was found to be associated with the type of fluoride exposure. Based on the prevalence of dental fluorosis and its effect on the intelligence of children, it appears that reducing fluoride levels in drinking water and monitoring water quality are important strategies for the prevention and treatment of dental fluorosis.

### Supplementary Information

Below is the link to the electronic supplementary material.

Supplementary file1 (DOC 254 kb)

## Data Availability

The datasets generated during and/or analyzed during the current study are available from the corresponding author on reasonable request.
