# Supplementary material for: Epidemiological analysis of drinking water-type fluorosis areas and the impact of fluorosis on children’s health in the past 40 years in China
Source: Environ Geochem Health. 2023 Oct 31;45(12):9925–40. doi: 10.1007/s10653-023-01772-9 (PMC10673999; doi:10.1007/s10653-023-01772-9)
Supplement: Supplementary file 1 — Supplementary file1 (DOC 254 kb) [file 10653_2023_1772_MOESM1_ESM.doc]

Table S1. Characteristics of the included studies.

| Name | Publication year | Survey time | Survey place | Water collection method | | Selected area | Water improving period (y) |
| --- | --- | --- | --- | --- | --- | --- | --- |
|  |  |  |  | Unimproved drinking water (village) | Improved drinking water (village) |  |  |
| Chen MS | 2000 | 1983-1996 | Hunan | East, south, west, north, center | Terminal piped water | / | 13 |
| Chen X | 2003 | 1991-2001 | Henan | East, south, west, north, center | Terminal piped water | / | 10 |
| Dong JM | 2011 | 1983-2006 | Jiangsu | East, south, west, north, center | Terminal piped water | 341villages | 23 |
| Guo SH | 2015 | 2015 | Shaanxi | East, south, west, north, center | Terminal piped water | 3 villages | 29 |
| Guo XC | 2002 | 2001 | Hunan | East, south, west, north, center | Terminal piped water | 10 counties /18 villages | 15 |
| He N | 2021 | 2009-2019 | Hebei | East, south, west, north, center | Terminal piped water | 598 villages | 10 |
| He XM | 2011 | 2008-2009 | Fujian | East, south, west, north, center | Factory water sample and terminal piped water | 4 villages | 24 |
| Huang WY | 2016 | 1987-2015 | Guangdong | East, south, west, north, center | Terminal piped water | 2 counties | 28 |
| Jia LH | 2011 | / | Hebei | East, south, west, north, center | Terminal piped water | 38 counties | / |
| Jian HH | 2013 | 2012 | Anhui | East, south, west, north, center | Terminal piped water | 2 counties /6 villages | / |
| Kan ZY | 2021 | / | Liaoning | East, south, west, north, center | Terminal piped water | 2279 villages | / |
| Kou BZ | 2015 | 2002- 2007 | Jilin | / | / | / | 6 |
| Li PF | 2014 | 2012-2013 | Shanxi | East, south, west, north, center | Terminal piped water | 6 counties | / |
| Li XJ | 2020 | 2018 | Xinjiang | East, south, west, north, center | Terminal piped water | 12 counties | 3 |
| Li YH | 2017 | / | Inner Mongolia | East, south, west, north, center | Terminal piped water | 30 villages | 5 |
| Liu ZH | 2019 | 2016-2017 | Tianjin | / | / | 524 villages | 15 |
| Lv FQ | 2014 | / | Gansu | East, south, west, north, center | Terminal piped water | / | 10 |
| Ma GQ | 2010 | 2009 | Henan | East, south, west, north, center | Terminal piped water | / | 30 |
| Ni N | 2020 | / | Jilin | East, south, west, north, center | Terminal piped water | / | 8 |
| Pang WL | 2011 | 1986-2009 | Zhejiang | East, south, west, north, center | Terminal piped water | 16 villages | 23 |
| Pu GL | 2019 | 2009- 2017 | Qinghai | East, south, west, north, center | Terminal piped water | 4 counties /12villages | 8 |
| Shi ML | 2011 | 1985-2009 | Xinjiang | East, south, west, north, center | Terminal piped water | / | 24 |
| Shu CL | 2013 | 2009 | Jiangsu | East, south, west, north, center | Factory water sample and terminal piped water | 3 cities /8 counties /24 villages | 30 |
| Sun GD | 2011 | 2004-2006 | Shandong | East, south, west, north, center | Terminal piped water | 11 villages | 3 |
| Wang JH | 2008 | 2005- 2007 | Liaoning | East, south, west, north, center | Factory water sample and terminal piped water | 13 cities /50 counties | 5 |
| Wang SQ | 2021 | / | Shandong | East, south, west, north, center | Terminal piped water | / | 5 |
| Xu XH | 2005 | 2003 | Shandong | East, south, west, north, center | Terminal piped water | 4 villages | 20 |
| Yu J | 2004 | 2002 | Guangdong | East, south, west, north, center | Terminal piped water | 3cities/24 villages | 15 |
| Yu QL | 2008 | 2004 | Gansu | / | / | 39 villages | 14 |
| Zhang JJ | 2022 | 1985-2000 | Inner Mongolia | East, south, west, north, center | Terminal piped water | / | 15 |
| Zhang M | 2010 | 1990-2010 | Hebei | East, south, west, north, center | Terminal piped water | / | 30 |
| Zhang N | 2019 | 1980-2016 | Liaoning | East, south, west, north, center | Terminal piped water | 42 villages | 36 |
| Zhang XF | 2012 | / | Tianjin | East, south, west, north, center | Terminal piped water | / | 10 |
| Zhao Y | 2015 | 2014 | Heilongjiang | East, south, west, north, center | Terminal piped water | 21 villages | / |
| Zhao YN | 2012 | 2011 | Ningxia | East, south, west, north, center | Terminal piped water | 11 villages | 5 |
| Zheng ZG | 2010 | 2009 | Guangxi | East, south, west, north, center | Factory water sample and terminal piped water | 3 villages | / |
| Zhi D | 2020 | 2018 | Liaoning | East, south, west, north, center | Terminal piped water | / | / |
| Chang ZL | 2018 | 2012- 2016 | Inner Mongolia | East, south, west, north, center | Terminal piped water | 11 counties /3 villages | 5 |

Table S1. (continued)

| Name | Publication year | Inclusion criteria | | | |
| --- | --- | --- | --- | --- | --- |
|  |  | Dental fluorosis | Urinary fluoride | Water fluoride | Skeletal fluorosis |
| Chen MS | 2000 | / | / | / | / |
| Chen X | 2003 | / | / | / | / |
| Dong JM | 2011 | Dean’s method | / | Standard test method for drinking water (GB/T5750.5-2006) | / |
| Guo SH | 2015 | Dean’s method | / | Standard test method for drinking water (GB/T5750.5-2006) | X-ray diagnosis of osteochondrosis (WS192-1999) |
| Guo XC | 2002 | Dean’s method | / | Standard test method for drinking water (GB5750-1985) | X-ray diagnosis of osteochondrosis (WS192-1999) |
| He N | 2021 | Industry standard of the Ministry of health (WS/T208-2011) | / | Standard test method for drinking water (GB/T5750.5-2006) | / |
| He XM | 2011 | Dean’s method | Ion-selective electrode method (WS/T89-1996) | Standard test method for drinking water (GB/T5750.5-2006) | X-ray diagnosis of osteochondrosis (WS192-1999) |
| Huang WY | 2016 | Industry standard of the Ministry of health (WS/T208-2011) | / | Standard test method for drinking water (GB/T5750.5-2006) | / |
| Jia LH | 2011 | Dean’s method | Ion-selective electrode method (WS/T89-1996) | Standard test method for drinking water (GB/T5750.5-2006) | Diagnostic criteria of endemic osteochondrosis (WS192-2008) |
| Jian HH | 2013 | Industry standard of the Ministry of health (WS/T208-2011) | / | Standard test method for drinking water (GB/T5750.5-2006) | / |
| Kan ZY | 2021 | / | / | Standard test method for drinking water (GB/T5750.5-2006) | / |
| Kou BZ | 2015 | Industry standard of the Ministry of health (WS/T208-2011) | Ion-selective electrode method (WS/T89-1996) | / | Diagnostic criteria of endemic osteochondrosis (WS192-2008) |
| Li PF | 2014 | Dean’s method | / | / | / |
| Li XJ | 2020 | Industry standard of the Ministry of health (WS/T208-2011) | Ion-selective electrode method (WS/T89-1996) | Standard test method for drinking water (GB/T5750.5-2006) | / |
| Li YH | 2017 | Dean’s method | / | Standard test method for drinking water (GB/T5750.5-2006) | / |
| Liu ZH | 2019 | Industry standard of the Ministry of health (WS/T208-2011) | / | Standard test method for drinking water (GB/T5750.5-2006) | / |
| Lv FQ | 2014 | Dean’s method | Ion-selective electrode method (WS/T89-1996) | Standard test method for drinking water (WS/T106-1999) | Clinical grade diagnosis of endemic osteochondrosis (GB16396-1996) |
| Ma GQ | 2010 | Dean’s method | Ion-selective electrode method (WS/T89-1996) | Standard test method for drinking water (GB/T5750.5-2006) | Diagnostic criteria of endemic osteochondrosis (WS192-2008) |
| Ni N | 2020 | Industry standard of the Ministry of health (WS/T208-2011) | / | / | / |
| Pang WL | 2011 | Dean’s method | / | Standard test method for drinking water (WS/T106-1999) | / |
| Pu GL | 2019 | Dean’s method | / | Standard test method for drinking water (GB/T5750.5-2006) | X-ray diagnosis of osteochondrosis (WS192-1999) |
| Shi ML | 2011 | / | / | / | / |
| Shu CL | 2013 | Dean’s method | Ion-selective electrode method (WS/T89-1996) | Standard test method for drinking water (GB/T5750.5-2006) | Diagnostic criteria of endemic osteochondrosis (WS192-2008) |
| Sun GD | 2011 | Dean’s method | Ion-selective electrode method (WS/T89-1996) | Standard test method for drinking water (WS/T106-1999) | / |
| Wang JH | 2008 | Dean’s method | Ion-selective electrode method (WS/T89-1996) | Standard test method for drinking water (GB/T5750.5-2006) | Clinical grade diagnosis of endemic osteochondrosis (GB16396-1996) |
| Wang SQ | 2021 | Industry standard of the Ministry of health (WS/T208-2011) | Ion-selective electrode method (WS/T 898-2015) | Standard test method for drinking water (GB/T5750.5-2006) | / |
| Xu XH | 2005 | Dean’s method | / | Standard test method for drinking water (GB5750-1985) | X-ray diagnosis of osteochondrosis (WS192-1999) |
| Yu J | 2004 | Dean’s method | / | Standard test method for drinking water (GB/T5750.5-2006) | X-ray diagnosis of osteochondrosis (WS192-1999) |
| Yu QL | 2008 | / | / | Standard test method for drinking water (GB5750-1985) | / |
| Zhang JJ | 2002 | Dean’s method | Ion-selective electrode method (WS/T89-1996) | Standard test method for drinking water (WS/T106-1999) | / |
| Zhang M | 2010 | Dean’s method | Ion-selective electrode method (WS/T89-1996) | Standard test method for drinking water (WS/T106-1999) | / |
| Zhang N | 2018 | Industry standard of the Ministry of health (WS/T208-2011) | / | Standard test method for drinking water (GB/T5750.5-2006) | / |
| Zhang XF | 2012 | / | Ion-selective electrode method (WS/T89-1996) | Standard test method for drinking water (GB/T5750.5-2006) | / |
| Zhao Y | 2015 | Industry standard of the Ministry of health (WS/T208-2011) | / | Standard test method for drinking water (GB/T5750.5-2006) | / |
| Zhao YN | 2012 | Dean’s method | / | Standard test method for drinking water (GB/T5750.5-2006) | / |
| Zheng ZG | 2010 | Dean’s method | Ion-selective electrode method (WS/T89-1996) | Standard test method for drinking water (GB/T5750.5-2006) | Diagnostic criteria of endemic osteochondrosis (WS192-2008) |
| Zhi D | 2020 | Industry standard of the Ministry of health (WS/T208-2011) | Ion-selective electrode method (WS/T 898-2015) | Standard test method for drinking water (GB/T5750.5-2006) | / |
| Chang ZL | 2018 | Industry standard of the Ministry of health (WS/T208-2011) | Ion-selective electrode method (WS/T 898-2015) | Standard test method for drinking water (GB/T5750.5-2006) | Diagnostic criteria of endemic osteochondrosis (WS192-2008) |

Table S1. (continued)

| **Name** | **Publication year** | **Age** | **Place** | **Selected area** | **Type** | **Method** | **Level** | **Water/Urine fluorine content** | |
| --- | --- | --- | --- | --- | --- | --- | --- | --- | --- |
|  |  |  |  |  |  |  |  | **Normal** | **Fluorosis** |
| Hong FG | 2001 | 8-14 | Shandong | School | Drinking water | CRT | Moderate | 0.75±0.34 | 2.9±1.17 |
| Jin X | 2020 | 7-12 | Tianjin | School | Drinking water | / | / | / | / |
| Li FH | 2009 | 8-12 | Hunan | School | Coal burning | CRT | Severe | 0.96±0.52 | 2.34±1.13 |
| Li XL | 2016 | 7-14 | Hubei | School | Coal burning | CRT | Severe | 0.93±074 | 2.45±1.25 |
| Li XS | 1995 | 8-12 | Henan | School | Drinking water | CRT | Moderate | / | / |
| Li YP | 2003 | 6-13 | Inner Mongolia | Country | Drinking water | CRT | Severe | / | / |
| Liu SS | 2000 | 10-12 | Tianjin | Country | Drinking water | CRT | Moderate | 0.37±0.3 | 3.15±0.7 |
| Liu XL | 1999 | 8-12 | Shaanxi | Country | Drinking water | / | Severe | 0.41±0.12 | 3.52±2.24 |
| Luo C | 2018 | 8-12 | Tianjin | Country | Drinking water | CRT | Moderate | 0.70±0.2 | 2.2±0.7 |
| Luo Y | 2018 | 8-12 | Guizhou | School | Coal burning | CRT | Severe | / | / |
| Ma Q | 2019 | 8-12 | Henan | School | Drinking water | CRT | Moderate | 0.81±0.34 | 2.13±0.74 |
| Mi P | 2019 | 7-12 | Tianjin | School | Drinking water | CRT | Mild | 0.31±0.32 | 1.55±1.2 |
| Wang GJ | 2012 | 8-13 | Jiangsu | School | Drinking water | CRT | Severe | 0.36±0.11 | 2.45±0.80 |
| Wang GJ | 2017 | 8-13 | Jiangsu | Country | Drinking water | CRT | Severe | 0.91±0.02 | 2.47±0.79 |
| Wang R | 2012 | 8-10 | Tianjin | School | Drinking water | CRT | Moderate | 0.90±0.09 | 2.82±0.17 |
| Wang R | 2021 | 9-11 | Hebei | Country | Drinking water | CRT | Severe | 1.0±0.07 | 2.8±0.06 |
| Wang S | 2012 | 8-12 | Jilin | School | Drinking water | CRT | Mild | / | / |
| Wang SX | 2005 | 8-12 | Shanxi | School | Drinking water | CRT | Severe | 0.48±0.23 | 8.31±1.85 |
| Wang SX | 2007 | 8-12 | Shanxi | School | Drinking water | CRT | Severe | 0.5±0.2 | 9.2±1.9 |
| Wang ZH | 2006 | 8-12 | Shanxi | School | Drinking water | CRT | Severe | 0.73±0.28 | 5.54±3.88 |
| Xiang J | 2019 | 8-13 | Jiangsu | School | Drinking water | CRT | Severe | 0.36±0.10 | 2.36±0.70 |
| Xiang Q | 2003 | 8-12 | Jiangsu | Country | Drinking water | CRT | Moderate | / | / |
| Yang SY | 1997 | 8-15 | Liaoning | Country | Drinking water | CRT | Mild | 0.4±0.1 | 2.1±0.5 |
| Yao LM | 1996 | 8-12 | Liaoning | Country | Drinking water | CRT | Severe | 0.5±0.2 | 10.1±1.2 |
| Yu LP | 2021 | 1-12 | Jilin | Country | Drinking water | CRT | Severe | 0.46±0.06 | 2.09±0.11 |
| Yu LY | 2014 | 8-10 | Tianjin | School | Drinking water | CRT | Severe | 0.85±0.23 | 3.1±2.1 |
| Yu X | 2018 | 8-12 | Tianjin | School | Drinking water | CRT | Moderate | 0.50±0.27 | 2.0±0.75 |
| Zhang JW | 1998 | 4-10 | Xinjiang | Country | Drinking water | CRT | Severe | / | / |
| Zhang PH | 2015 | 8-12 | Guizhou | Country | Drinking water | CRT | Severe | 0.83±0.71 | 3.32±1.02 |
| Zhang S | 2015 | 8-12 | Tianjin | School | Drinking water | CRT | Mild | 0.63±0.05 | 1.4±0.17 |
| Zhang XF | 2012 | 8-12 | Tianjin | School | Drinking water | CRT | Moderate | 0.63±0.07 | 1.4±1.3 |
| Dong L | 2018 | 8-12 | Shaanxi | School | Drinking water | CRT | Mild | 0.25±0.01 | 1.26±0.02 |

Table S2. Comparison of IQ scores of children with dental fluorosis in fluorosis areas.

| IQ Class | Test for heterogeneity | | Analysis model | Test for overall effect | | Odds Ratio | 95% CI | Fluorosis | Normal |
| --- | --- | --- | --- | --- | --- | --- | --- | --- | --- |
|  | *I*^2^ (%) | *P* |  | *Z* | *P* |  |  | Tolal/Events | Tolal/Events |
| ＜69 | 48 | = 0.14 | Fixed | 2.59 | = 0.010 | 1.58 | (1.12, 2.24) | 924/105 | 704/52 |
| 70-79 | 9 | = 0.33 | Fixed | 5.44 | < 0.00001 | 2.54 | (1.81, 3.55) | 924/153 | 704/50 |
| 80-89 | 34 | = 0.22 | Fixed | 2.51 | = 0.01 | 1.39 | (1.07, 1.79) | 924/196 | 704/113 |
| 90-109 | 0 | = 0.86 | Fixed | 4.26 | < 0.0001 | 1.55 | (1.27, 1.90) | 924/429 | 704/253 |
| 110-119 | 85 | = 0.001 | Fixed | 10.23 | < 0.00001 | 0.14 | (0.09, 0.20) | 924/35 | 704/162 |
| 120-129 | 28 | = 0.25 | Fixed | 6.22 | < 0.00001 | 0.08 | (0.04, 0.18) | 924/6 | 704/61 |
| ＞130 | 0 | = 0.94 | Fixed | 2.83 | = 0.005 | 0.09 | (0.02, 0.47) | 924/0 | 704/13 |
